# Services according to mental health needs for youth in foster care? – A multi-informant study

**DOI:** 10.1186/s12913-018-3365-6

**Published:** 2018-08-13

**Authors:** Marit Larsen, Valborg Baste, Ragnhild Bjørknes, Trine Myrvold, Stine Lehmann

**Affiliations:** 1 Regional Centre for Child and Youth Mental Health and Child Welfare –West, Uni Research Health, Bergen, Norway; 2 Uni Research Health, Bergen, Norway; 3 Department of Health Promotion and Development, Faculty of Psychology, University of Bergen, Bergen, Norway; 4 The Norwegian Institute for Urban and Regional Research, Oslo Metropolitan University, Oslo, Norway

**Keywords:** Foster youth, Service utilization, Mental health, Predictors, Multi-informant design

## Abstract

**Background:**

Foster children have a high risk of mental disorders. This has contributed to increased international attention to service utilization for youth in foster care. The aim of this study is to examine whether youth in foster care receive services according to need, by using a multi-informant design.

**Method:**

Detailed information on the type and frequency of service use during the last 2 years and on youth mental health were collected from foster youths and their carers in Norway (*n* = 405, aged 11–17 years) through online questionnaires. Mental health was assessed with the Strengths and Difficulties Questionnaire. Statistical analyses were conducted using descriptive statistics and log-binominal regressions.

**Results:**

In total, 48.8% of foster youths showed evidence of mental health problems, and 74.5% of foster families had contact with services. Increased mental health problems and living in non-kin foster care were associated with more service use. Youths with mental health problems had twice the probability of receiving services from the child and adolescent mental health service (CAMHS) and primary health care services compared to youths without problems. However, 57.0% of youths with carer-reported mental health problems did not have contact with CAMHS.

**Conclusions:**

Service use among foster youths was associated with service need rather than demographic and placement characteristics. The majority of youths with mental health problems did not receive services from CAMHS. However, many of them were in contact with primary health care services.

## Background

Youths in foster care are a highly vulnerable group. One in two foster children suffers from mental disorders [1], and comorbidity is high [1, 2]. These findings have contributed to increased attention to service utilization for youth in foster care [3, 4]. Knowledge about service utilization in this group relative to their need for services is essential to better understand the mechanisms of service access and ensure availability and the correct dimensioning of services. By using a multi-informant design, the present study examines mental health problems as an indicator of service need, and service utilization among foster youths in Norway. Further, we investigate whether contact with services is associated with youth mental health problems or demographic and placement characteristics.

Generally, children and youths in foster care have a high use of mental health services [5–9], also compared to the general youth-population [6, 7]. However, relative to their high rate of mental disorders, the service utilization by foster youth seems low, and findings indicate that a considerable part of this population does not receive services according to need [2, 10–12]. Much of the research on service utilization in foster care has used broad definitions of mental health services, in which different service providers are grouped together under this definition [5, 6, 13, 14]. Therefore, little knowledge exists about which specific services youths in foster care use. An exception is a Scottish study in which 60% of the foster youths (*N* = 192, aged 5–16 years) had mental health problems as measured by the Strengths and Difficulties Questionnaire (SDQ) [15]. These youths received a high level of service support from a wide range of agencies within the previous 6 months, with the exception of the child and adolescent mental health service (CAMHS). Social workers (93%) and general practitioners (55%) were the providers most often used. The study showed limited access to CAMHS, which has high competence in diagnostics and treatment. Two studies from the US have investigated special educational services [2, 11] and have yielded different rates of use of this service among foster children of 14.6% [11] and 52% [2].

There is a strong policy in Norway that individuals should receive services according to their need. According to an official Norwegian report, “*The health sector shall secure equal treatment based on health need, independent of personal economics, gender, ethnicity, residency, and the individuals living situation*” ([16], p. 29, our translation). Although demographic and placement characteristics are not representative of service need, such factors are related to service use among foster children. For example, having an ethnic minority background is related to lower service use in the US [2, 5, 9, 11], but not in Germany [12]. There are mixed findings regarding the relationship between gender and service utilization for foster children, with some studies finding that males use more services [7, 13], whereas others have found no relation between service use and gender [5, 6, 12]. Further, older age seems related to higher service utilization among foster children [5, 6, 13].

Regarding placement characteristics, living in kinship foster care is related to lower service utilization compared to living in non-kin foster care when controlling for mental health [9, 17]. Findings regarding placement stability and service use are inconclusive. A higher number of placement changes has been associated with higher service use [14], although another study found that a longer duration in foster care and more placement changes were related to a reduced likelihood of help seeking among foster children with ADHD [2].

Health needs should be related to service use, and in this article we use mental health problems as a proxy for service needs. The presence of more mental health problems has been found to be related to higher service use among foster children [2, 5, 12, 13, 15, 18]. In this group, higher service utilization has also been found to be especially related to externalizing problems [2, 6, 12] and to more complex symptom patterns and more severe mental health problems [13].

The prevalence and characteristics of mental health problems among children and youth vary depending on the type of informant [19]. In the general population, parents report more externalizing disorders, whereas adolescents themselves report more internalizing disorders [20]. Similarly, including youth self-reported SDQ scores to carer or teacher reports increased the identification of emotional disorders in foster youths, whereas relying only on youth reports increased the risk of overlooking conduct and hyperactivity problems [21]. This finding highlights the importance of using both carer- and youth reports when measuring youth mental health. However, most studies have used carer reports only when investigating the association between service use and youth’s mental health [12, 13].

Empirical studies of predictors of service use are ambiguous and scarce outside of the American context. There are substantial differences in the way Child Protective Services (CPS) are organized in different countries [22, 23]. In Norway, children are generally older when they are placed in foster care compared to the US, and adoption is rare [23]. Systematic knowledge of the type and frequency of service use among Norwegian foster youth and their families is lacking [24].

In this study, we first investigate youth mental health reported by carers and youths. Further, self- and carer reported frequency of contact with the following services is examined: CAMHS, primary health care (school health service, educational psychology service, general practitioner, and adolescent health clinic), CPS, special education, and “other services”. Second, we investigate whether the utilization of services from CAMHS and primary health care are associated with demographic characteristics (gender, age, and ethnicity), and placement characteristics (kinship foster care, and time in current foster home). Third, we investigate whether the utilization of services from CAMHS and primary health care are associated with self and carer-reported youth mental health (measured both dimensionally and dichotomous) and functional impairment.

## Methods

### Measures

Youth gender, age, and years living in the current foster home were derived from regional records in CPS and checked with the municipal CPS. Ethnicity of the child and kin/non-kin foster care were assessed through a purpose-made questionnaire to the carers. Youths were categorized as an ethnic minority if one or both biological parents were born in a non-western country. The foster home was defined as kinship care if the carer answered yes to the question “are you in biological family with the foster youth?”

In this study, mental health was measured with the Strengths and Difficulties Questionnaire (SDQ) [25]. This is a 25-item questionnaire for 4- to 17-year-olds measuring symptoms and impairments in the youth’s daily life. It may be completed by parents, teachers and as a self-report from the age of 11 years [21]. The SDQ has five subscales: Emotional Symptoms, Conduct Problems, Hyperactivity-Inattention, Peer Relationship Problems and Prosocial Behaviour. Each subscale consists of five items that are rated on a three-point scale (0–1-2), providing a total score range from 0 to 10. A Total difficulties score with a range from 0 to 40 is calculated by summing all four symptom subscales. The SDQ also contains an Impact scale comprising five items measuring distress and the interference of symptoms in the youth’s daily life [25]. This scale is referred to as a measure of functional impairment. The Impact score ranges from 0 to 10 for parent- and self-report. In this study, the SDQ was completed by youths and carers. The SDQ has been found to have satisfactory reliability and validity in general child populations [25, 26]. Structural validity for the five-factor model for the parent version of the SDQ was demonstrated when it was completed by Norwegian foster parents [27], and the predictive value of the carer-completed SDQ is supported for foster children [28]. The Emotional and Peer problems subscales were collapsed into an Internalizing subscale, and the Conduct and Hyperactivity-Inattention subscales were collapsed into an Externalization subscale, each with a score range of 0–20. These scales have been shown to have good convergent and discriminative validity [29] and have been used in previous studies of mental health in Norwegian general samples [30].

As recommended by Lehmann et al. [28], foster youths were considered to be in the clinical range for mental health problems with a score of 13 or higher on the foster parent-completed Total difficulties scale. Therefore, the Total difficulties scale was dichotomized as scores below the cut off = 0 and scores above/equal to the cut off =1.

Service use was measured through a custom made questionnaire asking how frequent the contact was with different services during the last 2 years. It was completed by all participating foster parents and by youth aged 13- to 17 years old. The youths were asked how often they had contact with different services, and carers were asked how often the youth (or themselves, for the youth) had contact with the services. The following seven services were included in the questionnaire: CAMHS, school health service, educational psychology service, general practitioner, adolescent health clinic, municipal CPS, and special education. The adolescent health clinic is a free service for youth aged 13 to 20. It provides counselling on sexual, mental and physical health questions. In addition, respondents were asked if they had contact with any other services, and were asked to name the service, if any, in an open textbox. For each type of service, the following categories of frequencies were listed: every week (= 4); every month (= 3); every 3 months (= 2); every 6 months (= 1); or more seldom/none at all (= 0). For each service, a Service Contact variable was made and coded yes (1) if the frequency category was 1 to 4 and no otherwise (0). It was coded separately for carers and youths. The variable Number of Services Used was calculated by summing Service Contact (0/1) for all services except CPS, yielding a score range from 0 to 7. Further, the variable Contact with Primary Health Care Services was defined as yes (= 1) if the respondent was coded yes on Service Contact on one or more of the four services: school health service, educational psychology service, general practitioner and adolescent health clinic.

### Procedure and study sample

The study was a part of the larger study, “Young in Foster Care”, within the research project “Children At Risk Evaluation (CARE) models”.

Data were collected between 1 October 2016 and 31 March 2017. Eligible foster youth were born between 1999 and 2005, had lived in their current foster home for at least 6 months following legally mandated placement and were placed by municipalities in the five counties encompassed by The Office for Children, Youth and family Affairs (Bufetat) – South (43 municipal CPS offices). Participants were assessed for eligibility from regional records from Bufetat South (*n* = 573) and from the municipal CPS (*n* = 279) in the same region. Heads of municipal CPS were asked to provide background information for all eligible youths. In total, 740 foster youth were identified as eligible.

Carers and youths were invited per postal mail with an information letter describing the study and how to complete the questionnaires, either through online completion on a secure webpage or by telephone interview. Foster mothers, foster fathers and youths were asked to complete the questionnaires separately. In accordance with Norwegian legislation, invitations to youths aged 11–15 years were placed in the letter addressed to the carers, whereas youths aged 16 and older received their information letter directly. Reminders were given by post and subsequent telephone contact. Through this telephone contact, additional 16 youths and four carers were identified as ineligible to participate. The youths were compensated with a gift card of 300 NOK (approximately 38 USD) for their participation. Carers were not compensated.

**Fig. 1:**
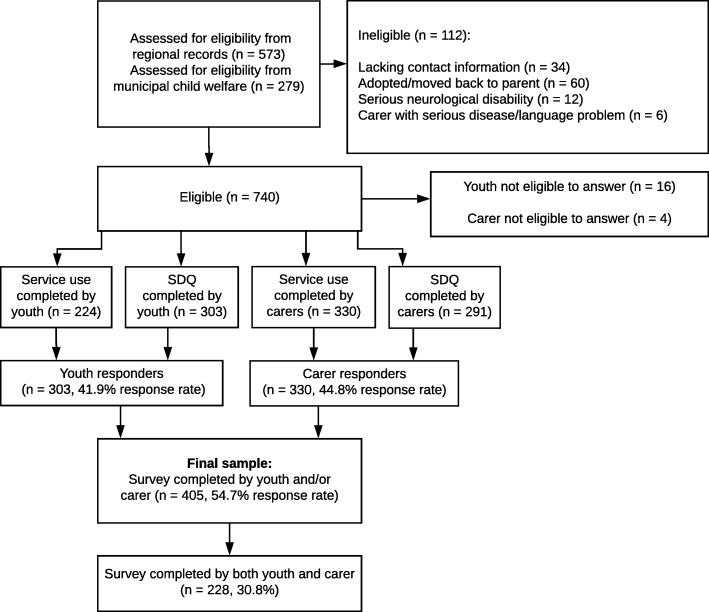
Flow-chart of data collection

The final sample consisted of 405 foster youths (54.7% response rate) with a response from a carer (330), youth (303), or both. Figure 1 provides a flowchart of the data collection and sample size for the different questionnaires. We combined foster fathers (*n* = 120) and foster mothers (*n* = 285) into one group of informants as there were no significant differences between foster mothers and foster fathers on reported service utilization and the SDQ Total difficulties scores. We prioritized information from the foster mother when available as most carer responders were foster mothers. This group is hereafter referred to as the “carers”. As only youths aged 13–17 were asked to answer the service use questionnaire 224 youths completed this.

### Ethics

The Regional Committee for Medical and Health Research Ethics, Western Norway approved the study. The Norwegian Directorate for Children, Youth and Family Affairs provided exemptions from confidentiality for caseworkers and carers. In accordance with the Norwegian ethics requirement, oral assent is required from children aged 12 years or older. The youths were instructed in their invitation letters that they could inform their carers if they did not want them to participate in the study.

### Data analysis

All descriptive analyses were conducted using IBM SPSS 24, while all log binominal regressions were conducted using STATA 15. The significance level was set to 0.05. Demographic and placement variables and Service Contact were presented as percentages, means, standard deviations (SD), and minimum and maximum values. Chi-square and t-tests were conducted to compare responders with non-responders on gender, age, and years in current foster home. For the SDQ scales, the means, SD, minimum and maximum scores, and Cronbach's alpha were calculated for carers and youths. The percentage above the cut off (> = 13) on SDQ Total difficulties was calculated for carers. For cases where both carers and youths had completed the SDQ, paired t-tests were used to compare carer and youth reports on all four SDQ scales. Similarly, McNemar tests were conducted to compare the Service Contact variables for youth and carer pairs for each service. As there were no differences between carers and youth Service Contact for CAMHS or any of the primary health care services, we used carer responses as indicators of service use in the further analyses.

Possible associations between demographic and placement variables and service use, were examined by log-binomial regressions with carer-reported CAMHS Contact and Contact with Primary Health Care Services (no =0, yes =1) as dependent variables. The independent variables were tested separately and included gender, age, ethnicity, kinship foster care, and years in current foster home. The results are presented with relative risk (RR) and a 95% confidence interval (CI). Any variable significantly associated with a service provider was also analysed with adjustments for dichotomized Total difficulties scores. To check for possible different predictive values between specific primary health care services and thereby to evaluate the validity of grouping them together, we conducted post hoc log-binominal regressions for each of the primary health care services (yes/no).

Possible associations between youth mental health and service use were examined by conducting log-binomial regressions with CAMHS Contact and Contact with Primary Health Care Services as dependent variables. The independent variables were tested separately and included both carer- and youth-completed SDQ Internalization and Externalization subscales, Impact scale, and dichotomized Total difficulties scores. To prevent unstable estimates due to a small number of youths for some scale scores, Internalization and Externalization subscale scores and the Impact scale score were recoded into broader score categories. All three scales started with zero, and then two and two scores were combined (e.g., scores 1 and 2 were collapsed into one category “1–2”, 3 and 4 into “3–4” and so on). Due to empty cells in the highest categories in the Internalization and Externalization subscales, scores from 15 and up were collapsed into one single category. Thus, the original 20 steps in the Internalization and Externalization subscales were reduced to 9 categories, and the original 10 steps in the impact scale were reduced to 6 categories. The scales were treated as continuous variables, and the results are presented with RR and 95% CI. Post hoc analyses of the association between the use of each of the primary health care services (yes/no) and mental health were conducted using log-binominal regressions. Further, post hoc log-binominal regressions were conducted to investigate possible associations between youth-completed SDQ scales and youth-reported CAMHS use.

## Results

Of the total study sample (*n* = 405), 56.1% were boys (*n* = 226). The mean age was 14.7 (SD = 2.02, range 11–17) and mean years in the current foster home was 6.7 (SD = 4.34, range 0.7–17.6). Of the 330 youths were carers have provided information about ethnicity and type of foster care, 23.9% (*n* = 79) were classified as an ethnic minority and 15.2% (*n* = 50) lived in kinship foster care. Drop-out analyses showed no differences between carer responders (*n* = 330) and non-responders (*n* = 410) on youth gender, age, and years in current foster home. Further, no differences were found between youth responders (*n* = 303) and non-responders (*n* = 437), with the exception of a higher mean age for responders compared to non-responders (14.8 years vs 14.3 years, *p* ≤ .001).

### Youth mental health

Table 1 shows the mean sum scores on the carer- and youth-completed SDQ Internalizing, Externalizing, Total and Impact scales with the maximum scale scores and Cronbach’s alpha for each scale. The internal consistency of the SDQ scales was acceptable to good. Carer-reported impact scores were higher compared to the youths’ score (*n* = 209, *p* < .001), in the paired analyses. No differences were found between carer- and youth-reported internalization or externalization problems or total difficulties (*p* = .188; *p* = .250; *p* = .157). A Total difficulties score above the cut off was reported by 48.8% of the carers.

**Table 1:** Scores on carer- and youth completed Strengths and Difficulties Questionnaire (SDQ)

		Sum score				
	N	Mean	SD	Min	Max	Cronbach’s alpha
Carer reported SDQ						
Internalizing problems	291	5.7	4.1	0	18	.78
Externalizing problems	291	7.0	4.2	0	18	.82
Total difficulties	291	12.7	7.2	0	33	.86
Impact score	291	2.7	2.9	0	10	.78
Youth reported SDQ						
Internalizing problems	303	5.4	4.0	0	16	.81
Externalizing problems	303	6.6	3.6	0	16	.78
Total difficulties	303	12.0	6.6	0	32	.85
Impact score	303	1.3	1.9	0	8	.87

Note: Subscales mean, and minimum and maximum of sum scores

### Service utilization

Table 2 presents the frequency of service utilization. Table 3 shows service contact and frequency of use for each service, reported by carers and youth separately. Overall, 74.5% of carers and 68.7% of youths reported contact with any service. Contact with CAMHS was reported by 31.2% of carers and 27.2% of youth. Further, 61.2% of carers and 58.5% of youth reported Contact with Primary Health Care Services. CPS stands out as the single service most used by carers and youths; 92.1 and 85.3%, respectively, reported having any contact. The second most used service was special education (41.7%), reported by carers, and the school health service (30.8%), reported by youth. The only differences in reported Service Contact (yes/no) when comparing youth and carer responders on the same case, were in special education (*p* = .008) and other services (*p* = .016), with carers reporting more service use.

**Table 2:** Carer and youth reported number of different services used

Number of Services Used^a^	N	n	Percent	Mean	SD	Min	Max
Carer reported	330			1.90	1.61	0	7
0 services		84	25.5				
1–2 services		138	41.8				
3–4 services		86	26.1				
5–7 services		22	6.7				
Youth reported	224			1.54	1.53	0	7
0 services		70	31.3				
1–2 services		98	43.8				
3–4 services		45	20.1				
5–7 services		11	4.9				

Note: ^a^Summed Service Contact scores for all services, except CPS

**Table 3:** Service Contact Reported by carers (*n* = 330) and youths (*n* = 224)

	Service Contact	Distribution of use for the ones that have had contact				
		Every week (4)	Every month (3)		Every 3. Month (2)	Every 6. Month (1)
	% (*n*)	% (*n*)	% (*n*)		% (*n*)	% (*n*)
CAMHS						
Carers	31.2 (103)	23.3 (24)	43.7 (45)		13.6 (14)	19.4 (20)
Youth	27.2 (61)	18.0 (11)	44.3 (27)		16.4 (10)	21.3 (13)
Contact with Primary Health Care services:						
Carers	61.2 (202)					
Youth	58.5 (131)					
School Health service						
Carers	27.6 (91)		5.5 (5)	20.9 (19)	28.6 (26)	45.1 (41)
Youth	30.8 (69)		14.5 (10)	15.9 (11)	33.3 (23)	36.2 (25)
Educational Psychology Service						
Carers	34.8 (115)		5.2 (6)	10.4 (12)	34.8 (40)	49.6 (57)
Youth	19.6 (44)		11.4 (5)	27.3 (12)	15.9 (7)	45.5 (20)
General Practitioner						
Carers	29.7 (98)		1.0 (1)	6.1 (6)	27.6 (27)	65.3 (64)
Youth	35.7 (80)		2.5 (2)	10.0 (8)	30.0 (24)	57.5 (46)
Adolescent Health Clinic						
Carers	7.3 (24)		0 (0)	20.8 (5)	8.3 (2)	70.8 (17)
Youth	11.6 (26)		3.9 (1)	11.5 (3)	19.2 (5)	65.4 (17)
Other service providers:						
Municipal CPS						
Carers	92.1 (304)		2.3 (7)	20.4 (62)	54.9 (167)	22.4 (68)
Youth	85.3 (191)		1.0 (2)	7.9 (15)	52.4 (100)	38.7 (74)
Special Education						
Carers	42.7 (141)		77.3 (109)	9.2 (13)	3.6 (5)	9.9 (14)
Youth	21.9 (49)		65.3 (32)	12.2 (6)	12.2 (6)	10.2 (5)
Other services						
Carers	16.4 (54)		22.2 (12)	38.9 (21)	22.2 (12)	16.7 (9)
Youth	7.1 (16)		18.8 (3)	31.3 (5)	25.0 (4)	25.0 (4)

Note: *CAMHS* child and adolescent mental health service. Primary health care services include: the school health service, educational psychology service, general practitioner, and the adolescent health clinic, *Municipal CPS* Municipal Child Protective Service

### Associations between demographic and placement characteristics and service use

No demographic or placement variables were associated with having contact with CAMHS. Kinship foster care was associated with decreased use of the primary health care services (RR = 0.68, 95% CI [0.50, 0.95]) (Table 4). When controlling for dichotomized Total difficulties score, this association was still significant (RR = 0.65, 95% CI [0.45, 0.95]). Post hoc analyses of each primary health care service revealed that girls used the school health service (RR = 2.03, 95% CI [1.41, 2.92]) and the adolescent health clinic (RR = 3.14, 95% CI [1.34, 7.37]) more than boys did. In contrast, boys used the educational psychology service (RR = 1.40, 95% Cl [1.02, 1.91]) more than girls did. For this service, more time in the current foster home was also associated with more use (RR = 1.04, 95% Cl [1.01, 1.07]).

**Table 4:** Associations between CAMHS and Primary Health Care Service Contact, and demographic- and placement characteristics

			CAMHS utilization			Primary Health Care Service utilization		
		*n*	%	RR	95% CI	%	RR	95% CI
Gender	Female	143	35.0	1.00		62.9	1.00	
	Male	185	28.1	0.80	[0.58, 1.11]	60.0	0.95	[0.80, 1.13]
Age (years)		330		0.98	[0.91, 1.07]		1.01	[0.96, 1.05]
Ethnicity	Majority	251	28.7	1.00		61.4	1.00	
	Minority	79	39.2	1.37	[0.98, 1.92]	60.8	0.99	[0.81, 1.21]
Type of foster care	Non kin	280	30.0	1.00		64.3	1.00	
	Kin	50	38.0	1.27	[0.85, 1.88]	44.0	**0.68**	**[0.50, 0.95]**
Years in current foster home		330		0.98	[0.95, 1.02]		1.00	[0.98, 1.02]

Note: RR = relative risk; CI = confidence interval. Log-binominal regression with CAMHS and Primary Health Care Service utilization (No =0, Yes =1) as dependent variables, separate models for each independent variable. Primary Health Care include the following services: school health service, educational psychology service, general practitioner, and adolescent health clinic. Significant associations are marked in **boldface**

### Associations between youth mental health and service use

Increased carer-reported internalizing and externalizing problems and functional impairment were associated with increased use of CAMHS and primary health care (Table 5). Further, Total difficulties scores above the cut off doubled the probability of being in contact with CAMHS (RR = 2.00, 95% CI [1.39, 2.87]) and primary health care (RR = 1.82, 95% CI [1.48, 2.23]) compared to scores below the cut off. Among youths who had scores above the cut off, 43.0% of the carers reported contact with CAMHS, and 78.2% with primary health care, during the last 2 years.

**Table 5:** Associations between CAMHS and Primary Health Care Service Contact and youth mental health

			CAMHS utilization			Primary Health Care Service utilization		
		*n*	%	RR	95% CI	%	RR	95% CI
Carer reported mental health								
Internalizing problems		291		**1.22**	**[1.15, 1.30]**		**1.11**	**[1.08, 1.13]**
Externalizing problems		291		**1.10**	**[1.02, 1.19]**		**1.11**	**[1.07, 1.15]**
Impact		291		**1.19**	**[1.08, 1.31]**		**1.18**	**[1.13, 1.25]**
Total difficulties^a^	Below	149	21.5	1.00		43.0	1.00	
	Above	142	43.0	**2.00**	**[1.39, 2.87]**	78.2	**1.82**	**[1.48, 2.23]**
Youth reported mental health								
Internalizing problems		228		1.09	[1.00, 1.19]		**1.09**	**[1.05, 1.13]**
Externalizing problems		228		1.02	[0.92, 1.13]		**1.08**	**[1.03, 1.13]**
Impact		228		1.11	[0.94, 1.32]		**1.13**	**[1.05, 1.21]**

Note: *RR* relative risk, *CI* confidence interval. Log-binominal regression with CAMHS and Primary Health Care Service utilization (No =0, Yes =1) as dependent variables, separate models for each independent variable. Primary Health Care includes the following services: school health service, educational psychology service, general practitioner, and adolescent health clinic. Mental health is measured with the Strength and Difficulties Questionnaire

^a^Total difficulties: below and above cut off

Significant associations are marked in **boldface**

Increased youth-reported internalizing and externalizing problems and functional impairment were associated with increased use of primary health care services. There were no relations between youth-reported mental health or functional impairment and carer-reported CAMHS use. However, post hoc analyses of youth-reported contact with CAMHS showed that there were positive associations between youth reported CAMHS utilization and youth-reported internalizing problems (RR = 1.20, 95% CI [1.10, 1.31]), externalizing problems (RR = 1.12, 95% CI [1.01, 1.24]), and functional impairment (RR = 1.38, 95% CI [1.21, 1.58]).

The post hoc analyses of each primary health care service separately showed that youth-reported internalizing and externalizing problems, and functional impairment were not associated with general practitioner contact, and youth-reported functional impairment was not associated with the use of the adolescent health clinic. Carer-reported youth internalizing and externalizing problems, functional impairment and dichotomized total difficulties were associated with increased use of all primary care services except for the adolescent health clinic.

## Discussion

Of the foster youths in our sample, 48.8% had a total difficulties score indicative of mental health problems. There was a high prevalence of service use, with 31.2% reporting contact with CAMHS and 61.2% with primary health care services during the last 2 years. Living in kinship foster care was associated with lower use of primary health care services. No other demographic or placement characteristics were related to contact with CAMHS or primary health care services. Youth mental health problems were related to more contact with both service providers. Youths with Total difficulties scores above cut off had a doubled probability of contact with both CAMHS and primary health care services compared to those scoring below the cut off. However, more than half of the youths with indications of mental health problems had not received services from CAMHS during the last 2 years.

The finding that 48.8% of youths showed indications of mental health problems is in accordance with results from a recent meta-analysis including studies from 5 different Western countries, which found that 49% of children in the child welfare system qualify for a mental disorder [31]. The only difference when comparing carer and youth scores on the SDQ scales was on reported functional impairment, with carers reporting that youths’ mental health problems had a larger impact on the youths’ daily lives. This finding contrasts with earlier studies that have found that youths report more internalizing problems, whereas carers and parents report more externalizing problems [20, 21].

Our finding that 68.7% of youths reported contact with any help services, excluding CPS, is in line with results from a Norwegian study on youth in residential care (*n* = 400, aged 12–20) [32] in which 60.6% of youths reported contact with any services for mental health problems during the last 3 months. In the general youth population, 6.9% have sought help from different services for mental health problems during the last year [33], which is substantially lower than our findings. These results show that foster youth have a higher incidence of overall service use compared to the general Norwegian population, which are in line with higher estimates of mental disorders in foster youths compared to the general population [1].

Between 27.2 and 31.2% of the foster youth had contact with CAMHS during the last 2 years. This percentage is high compared to findings from other studies on this group [10, 15]. In the study by Minnis et al. [15], 18% of the foster children had contact with CAMHS. One possible explanation may be that Norway has an extensive welfare system, and therefore CAMHS might be more readily available. As higher age is related to more service use [5, 6, 13], it is also possible that our higher rate of CAMHS contact is due to a higher age range in our sample. However, our results for CAMHS use were low compared to other studies [11, 13], which may be a consequence of our narrower definition of CAMHS, whereas other studies have placed several different service providers under this definition.

We found that the largest service provider was CPS, with which 92.1% of carers reported having contact. The most frequent answer regarding the frequency of contact was “every third month” for both carers and youth, which is in line with the Norwegian legislation that municipal CPS is obliged to have contact with the foster family at least four times each year [34]. However, our findings that some families have no contact with CPS, indicates a divergence between legally stated rights and actual follow up for some families. However, a considerable part of the group (22.7%) reported contact with CPS each month or more often. Taken together, our findings indicates substantial variations in follow-up from CPS, with some families receiving extra follow up while others do not receive the contact to which they legally have a right.

Special education was the second most used service reported by carers, with 42.7% of the youth receiving this service. This finding is in accordance with the finding that 52% of foster children in the US use special education [2]. However, only 21.9% of the youths in our sample reported receiving special education. We may only speculate, but this finding could indicate that many of the youths are not aware of the special education they receive in school. This may be problematic as youths should be heard in decisions regarding their own treatment, which is difficult if they are not aware of which services they receive.

Overall, 61.2% of carers and 58.5% of youth reported contact with primary health care services. As the organization of services varies, it is difficult to compare service use from multiple providers between different countries. However, our results are in line with findings that foster youth receive a high level of services from a wide range of agencies [15]. The fact that one third of our sample was in contact with three or more different services highlights the importance of coordination and collaboration between services to provide adequate and coherent services for youth in foster care.

Youths in kinship foster care had less contact with primary health care services compared to youths in non-kin foster care, even when adjusting for mental health. This finding is in line with earlier research [9, 17] and indicates that the association is not explained by youths in kinship foster care having fewer mental health problems. It is surprising that no other demographic or placement characteristics were related to service use as other studies indicate that these factors have an impact (e.g., [2, 5, 12, 13, 35]).However, post hoc analyses of each of the primary care services separately nuanced these results. Girls had more contact with the school health service and the adolescent health clinic, whereas boys had more contact with the educational psychology service. These results corresponds with findings from the general Norwegian population [36]. Our findings suggest that boys and girls in foster care use different services, although at overall similar rates. Differences in the types of service used can stem from boys and girls having different types of problems; thus, different types of services are suited to their needs. However, our findings indicate that mostly girls use the services that are directly available for the youths themselves. This calls into question whether low threshold services are available for boys or designed in a way that they will use them. However, our results from the post hoc analyses must be interpreted with caution given the increased likelihood of type 1 errors with multiple testing.

Carer-reported internalizing and externalizing problems, total difficulties, and functional impairment were all related to CAMHS and primary health care use. Our results do not indicate that externalizing problems have a higher predictive value for receiving services compared to internalizing problems. This contrasts with earlier findings suggesting that externalizing difficulties are more closely related to service use than internalizing problems are among foster youths [2, 6, 12] and in the general population [37]. Our results are more consistent with findings that foster children with more severe difficulties have higher service use, with no differences in service access between types of mental health problems [13].

Even though youths with indications of mental health problems had twice the probability of being in contact with CAMHS and primary health care services, more than half of this group did not have contact with CAMHS. This could indicate an underuse of specialized mental health services among foster youths. However, 78.2% of youths with mental health problems were in contact with different primary health care services. Among institutionalized Norwegian youth, 37.8% had contact with CAMHS during the last 3 months [32]. In this group, less than 50% of those with mental disorders received help from CAMHS, whereas two-thirds received help from primary health care and special education. Combined, these results suggest that primary health care services, rather than CAMHS, is the main service provider for both institutionalized and foster youth with mental health problems.

Further, whereas youth-reported mental health problems were associated with the use of primary health care services, this was only associated with self-reported, not carer-reported, contact with CAMHS. Small differences between youth and carers in reported CAMHS use are expected as carers may receive supervision from CAMHS without the youth having direct contact. From the age of 16, youths may receive services from CAMHS without the carer’s assent or knowledge [38]. The finding that the strength of association between mental health and service use depend on informant used, highlight the value of using multiple informants on both variables when investigating the association between measures of mental health and service utilization.

### Strengths and limitations

This study has the advantage of using a multi-informant design with information from both carers and youths regarding mental health and service use. Further, we provide detailed information about contact with eight different services and frequency of service contact. Another strength of our study is that our sample seems representative of the general foster care population [39], even though our percentage of responders living in kinship foster care was somewhat low (15.2% versus 25%) [39].

One limitation of this study is that we have a two-year recall period of service use, which can be challenging to remember correctly, especially for younger youths. Further, we ask the participants to differentiate between several service providers, which might be challenging for the respondents. However, as there were few significant differences between youth and carer reported service contact for each service, this can indicate that the youths have a similar understanding to their carers with regards to which services they’ve had contact with during the last 2 years. Further, we lack information about reasons for contact with the different services. Thus, we do not know how much of the contact targeted mental health problems as opposed to contact for other reasons, such as somatic health problems or learning difficulties. However, findings from the general Norwegian population show that high proportions (76 -77%) of youth in contact with the school health service, adolescent health clinic and educational psychology service show evidence of mental health problems [36]. Further, the main reason for contact with the general practitioner is mental health problems for youth aged 15–24 [40]. These findings indicates that mental health problems are a prevalent focus in contact with these services. However, this is less of a limitation when investigating contact with CAMHS as this is a specialized service targeting mental health problems.

Because the cut-off value for SDQ Total difficulties was derived from a study on foster children aged 6–12 years old [28], there is uncertainty about the validity of using this cut off in our group of older foster youth. However, a Swedish study of 13-year-olds in the general population found that norms for being in the 90th percentile on Total difficulties on the parent-completed SDQ were 13.0 for girls and 13.9 for boys [41], which are in line with our cut-off value.

## Conclusions

The present paper describes mental health, the type and frequency of service use, and factors associated with service utilization for 11- to 17-year-old foster youth in Norway. In our sample, 48.8% of youths had indications of mental health problems, and they had a high rate of service utilization from a wide range of services. Our findings indicate that service need, measured as mental health problems, rather than demographic and placement characteristics seems to have importance for service use. Even though youths with mental health problems had a doubled probability of receiving services, less than half of them had contact with CAMHS. As 78.2% of youths with mental health problems receive service support from primary health care services, it is possible that many have their service needs met there. To secure stepped care, screening procedures should be used in primary health care services to identify the youths in need for more specialized services. Further, as youths in foster care often are in contact with several service providers it is important to have a good collaboration between services.

We need more knowledge on foster youths’ and their carers’ experiences with services and whether they consider this contact helpful and suited to their needs. Lastly, as there is a lack of knowledge regarding whether services as presently offered are effective in reducing symptoms and increasing wellbeing in foster youth, future research on the effect of specified treatment approaches for foster youth is needed.

## Abbreviations

CAMHS: Child and adolescent mental health service
CI: Confidence interval
CPS: Child protective service
RR: Relative risk
SD: Standard deviations
SDQ: Strength and Difficulties Questionnaire
