# Correction to: Services according to mental health needs for youth in foster care? – a multi-informant study

**DOI:** 10.1186/s12913-018-3496-9

**Published:** 2018-09-11

**Authors:** Marit Larsen, Valborg Baste, Ragnhild Bjørknes, Trine Myrvold, Stine Lehmann

**Affiliations:** 1 Regional Centre for Child and Youth Mental Health and Child Welfare –West, Uni Research Health, Bergen, Norway; 2 Uni Research Health, Bergen, Norway; 3 Department of Health Promotion and Development, Faculty of Psychology, University of Bergen, Bergen, Norway; 4 The Norwegian Institute for Urban and Regional Research, Oslo Metropolitan University, Oslo, Norway

## Correction

Following publication of the original article [1], the authors reported a misaligning of data in Table 3 (Table 1 here) (weekly results have been put in the column for monthly results).

**Table 1:**
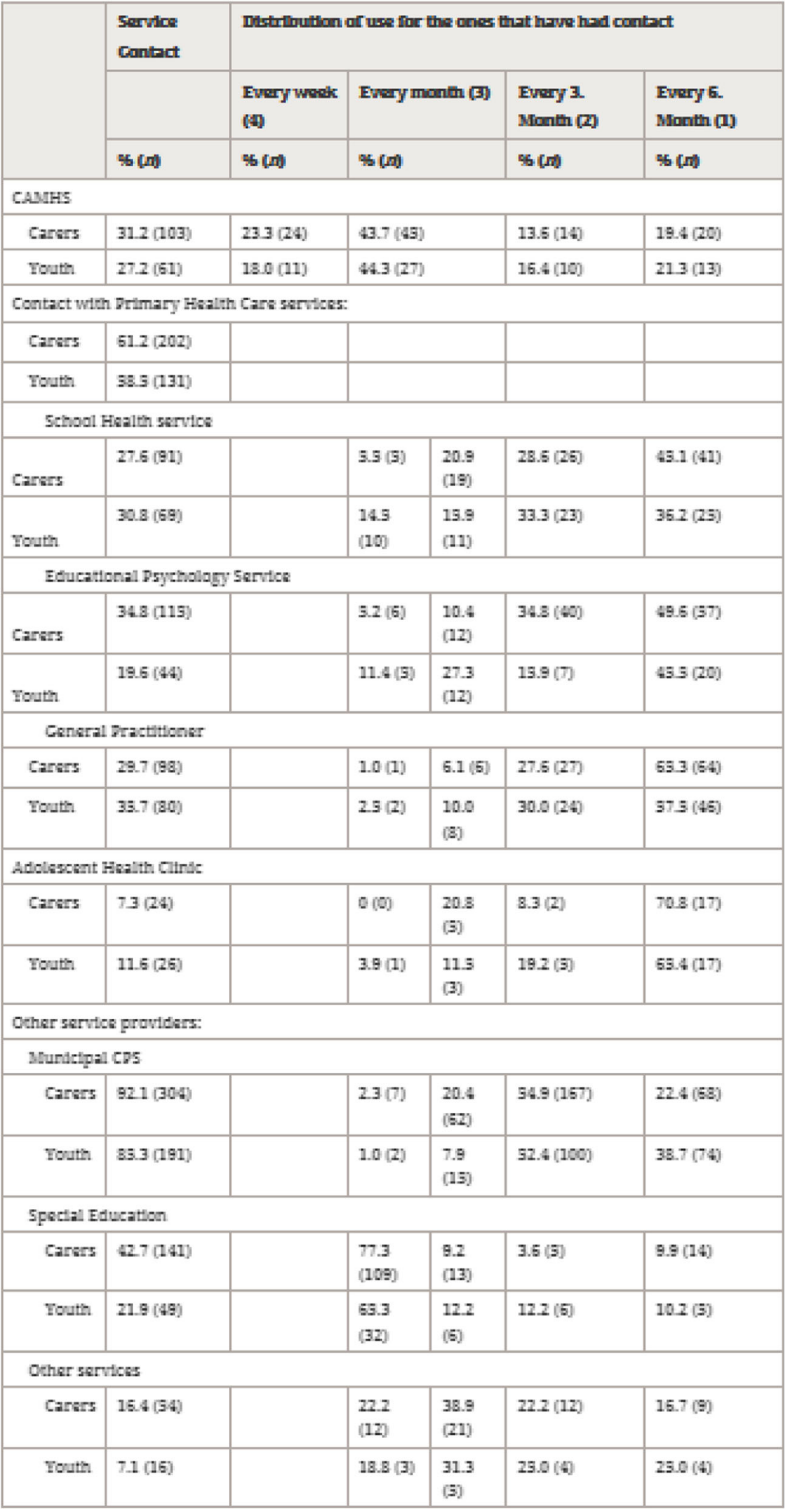
Service contact reported by carers (*n* = 330) and youths (*n* = 224)

**Table 2:** Service contact reported by carers (*n* = 330) and youths (*n* = 224)

	Service contact	Distribution of use for the ones that have had contact.			
		Every week (4)	Every month (3)	Every 3. month (2)	Every 6. month (1)
	% (n)	% (n)	% (n)	% (n)	% (n)
CAMHS					
Carers	31.2 (103)	23.3 (24)	43.7 (45)	13.6 (14)	19.4 (20)
Youth	27.2 (61)	18.0 (11)	44.3 (27)	16.4 (10)	21.3 (13)
Contact with primary health care services:					
Carers	61.2 (202)				
Youth	58.5 (131)				
School health service					
Carers	27.6 (91)	5.5 (5)	20.9 (19)	28.6 (26)	45.1 (41)
Youth	30.8 (69)	14.5 (10)	15.9 (11)	33.3 (23)	36.2 (25)
Educational psychology service					
Carers	34.8 (115)	5.2 (6)	10.4 (12)	34.8 (40)	49.6 (57)
Youth	19.6 (44)	11.4 (5)	27.3 (12)	15.9 (7)	45.5 (20)
General Practitioner					
Carers	29.7 (98)	1.0 (1)	6.1 (6)	27.6 (27)	65.3 (64)
Youth	35.7 (80)	2.5 (2)	10.0 (8)	30.0 (24)	57.5 (46)
Adolescent health clinic					
Carers	7.3 (24)	0 (0)	20.8 (5)	8.3 (2)	70.8 (17)
Youth	11.6 (26)	3.9 (1)	11.5 (3)	19.2 (5)	65.4 (17)
Other service providers:					
Municipal CPS					
Carers	92.1 (304)	2.3 (7)	20.4 (62)	54.9 (167)	22.4 (68)
Youth	85.3 (191)	1.0 (2)	7.9 (15)	52.4 (100)	38.7 (74)
Special Education					
Carers	42.7 (141)	77.3 (109)	9.2 (13)	3.6 (5)	9.9 (14)
Youth	21.9 (49)	65.3 (32)	12.2 (6)	12.2 (6)	10.2 (5)
Other services					
Carers	16.4 (54)	22.2 (12)	38.9 (21)	22.2 (12)	16.7 (9)
Youth	7.1 (16)	18.8 (3)	31.3 (5)	25.0 (4)	25.0 (4)

The correct Table 3 (Table 2 here) is shown below.

The original publication of this article has been corrected.
